# Pegvisomant in acromegaly: a multicenter real-life study in Argentina

**DOI:** 10.20945/2359-3997000000160

**Published:** 2019-08-14

**Authors:** Natalia Ximena Garcia Basavilbaso, Maria Carolina Ballarino, Darío Bruera, Oscar D. Bruno, Alberto B. Chervin, Karina Danilowicz, Patricia Fainstein-Day, Silvina Gabriela Fidalgo, Adriana Frigeri, Mariela Glerean, Rodolfo Guelman, Gabriel Isaac, Debora Adela Katz, Pablo Knoblovits, Fabiana Librandi, Monica López Montes, Maria Susana Mallea-Gil, Marcos Manavela, Paula Mereshian, Daniel Moncet, Analia Pignatta, Amelia Rogozinsky, Laura R. Sago, Marisa Servidio, Monica Spezzi, Graciela Stalldecker, Julieta Tkatch, Nicolas Marcelo Vitale, Mirtha Guitelman

**Affiliations:** 1 Departamento de Endocrinología Hospital Carlos G. Durand CABA Argentina Departamento de Endocrinología Hospital Carlos G. Durand, CABA, Argentina; 2 Hospital Militar Central CABA Argentina Servicio de Endocrinología Hospital Militar Central, CABA, Argentina; 3 Clínica Caraffa Clínica Caraffa Córdoba Argentina Servicio de Endocrinología Clínica Caraffa, Córdoba, Argentina; 4 Universidad de Buenos Aires Hospital de Clínicas “José de San Martín” Universidad de Buenos Aires CABA Argentina Servicio de Endocrinología Hospital de Clínicas “José de San Martín”, Universidad de Buenos Aires, CABA, Argentina; 5 Hospital Santa Lucía Hospital Santa Lucía CABA Argentina Servicio de Endocrinología Hospital Santa Lucía, CABA, Argentina; 6 Hospital Italiano Hospital Italiano CABA Argentina Servicio de Endocrinología Hospital Italiano, CABA, Argentina; 7 Hospital Churruca CABA Argentina Servicio de Endocrinología Hospital Churruca, CABA, Argentina; 8 Hospital Teodoro Alvarez CABA Argentina Servicio de Endocrinología Hospital Teodoro Alvarez, CABA, Argentina; 9 Hospital Privado de la Comunidad Mar del Plata Argentina Servicio de Endocrinología Hospital Privado de la Comunidad, Mar del Plata, Argentina; 10 CABA Argentina Servicio de Endocrinología Fleni, CABA, Argentina; 11 Hospital Rivadavia CABA Argentina Servicio de Endocrinología Hospital Rivadavia, CABA, Argentina,; 12 Universidad Nacional de Córdoba Hospital Clínicas Universidad Nacional de Córdoba Córdoba Argentina Servicio de Endocrinología Hospital Clínicas, Universidad Nacional de Córdoba, Córdoba, Argentina; 13 Hospital Interzonal San Juan Bautista La Rioja Argentina Servicio de Endocrinología Hospital Interzonal San Juan Bautista, La Rioja, Argentina; 14 Hospital Ramos Mejía CABA Argentina Servicio de Endocrinología Hospital Ramos Mejía, CABA, Argentina,; 15 Hospital Italiano de La Plata La Plata Argentina Servicio de Endocrinología Hospital Italiano de La Plata, La Plata, Argentina,; 16 Instituto Médico Platense Mar del Plata Argentina Servicio de Endocrinología Instituto Médico Platense, Mar del Plata, Argentina; 17 CABA Argentina Servicio Hospital. Pirovano, CABA, Argentina

**Keywords:** Pegvisomant, acromegaly, GH receptor antagonist, clinical trial

## Abstract

**Objective:**

To describe the long term safety and efficacy of pegvisomant (PEGV), and the predictors of treatment response in patients with acromegaly in the real life setting.

**Subjects and methods:**

We retrospectively reviewed the clinical, hormonal and radiological data of acromegalic patients treated with PEGV in 17 Argentine centers.

**Results:**

Seventy-five patients (age range 22-77, 51 females) with acromegaly have been treated with PEGV for up to 118 months (median 27 months). Before PEGV, 97.3% of patients had been treated with medical therapy, surgery and/or radiotherapy, two patients had no previous treatment. At that time, all patients had an IGF-1 above the upper normal limit (ULN) (mean 2.4 x ULN ± 0.98, range 1.25-7). At diagnosis of acromegaly 84% presented macroadenomas, prior to PEGV only 23,5% of patients remained with tumor remnant > 1 cm, the remaining showed normal or less than 1 cm images. Disease control (IGF-1 ≤ 1.2 x ULN) was achieved in 62.9% of patients with a mean dose of 11.8 mg/day. Thirty-four patients (45%) received PEGV monotherapy, while 41 (55%) received combined therapy with either somatostatin analogues and/or cabergoline. Adverse events related to PEGV were: local injection site reaction in 5.3%, elevated liver enzymes in 9.3%, and tumor size growth in 9.8%. Pre-PEGV IGF-I level was the only predictor of treatment response: 2.1 x ULN vs 2.8 x ULN in controlled and uncontrolled patients respectively (p < 0.001).

**Conclusion:**

this long term experience indicates PEGV treatment was highly effective and safe in our series of Argentine patients with acromegaly refractory to standard therapies. Arch Endocrinol Metab. 2019;63(4):320-7

## INTRODUCTION

Acromegaly is a chronic disease characterized by excessive secretion of growth hormone (GH), most often from a growth hormone-secreting pituitary adenoma, with resultant hepatic overproduction of insulin-like growth factor I (IGF-١) (1). It is a rare condition, with an estimated incidence of 3 to 11 cases per million inhabitants per year (2) and a prevalence of 40 to 78 cases per million inhabitants (3). GH excess is associated with a significant increase in morbidity, including hypertension, diabetes, cardiovascular disease, sleep apnea, and cancer. Reduction of GH levels and normalization of IGF-1 reduces mortality to rates similar to those reported in the general population (4-6).

The treatment of choice for most patients is transsphenoidal surgery, with results depending on tumor characteristics and neurosurgeon’s experience. Experienced surgeons can achieve a cure rate of up to 90% in patients with microadenomas and of 50% to 70% in macroadenomas (7,8). Approximately half of patients will not be cured by surgery and will require adjuvant medical therapy and/or radiotherapy. Three classes of drugs are currently available: Somatostatin receptor ligands (SRLs), dopamine agonists (DA) and GH receptor antagonist, PEGV (9).

The first-generation SRLs, octreotide and lanreotide, are the drugs of choice in adjuvant therapy of acromegaly when remission has not been achieved after surgery as well as while awaiting the effect of radiotherapy. The rate of IGF-1 normalization with SRLs in patients who were naïve to medical therapy as well as in those who have undergone surgery ranges between 38% and 68% (10-12).

Pasireotide, a second-generation SRL approved in 2014 for use in acromegaly is effective in 15%-20% of patients who are not controlled using first-generation SRLs, at doses of 40 mg and 60 mg, respectively. However, the higher rates of hyperglycemia and diabetes might affect its use in some cases (13).

Cabergoline a D2 receptor agonist, achieves IGF-1 normalization in up to 34% and 50% of cases as monotherapy or combined therapy with SRL respectively (14).

PEGV, a genetically modified analog of human GH, is the only drug acting as a GH receptor antagonist that, when binding to this receptor, inhibits IGF-1 synthesis and release (15). This drug was approved in 2003 by the FDA for the treatment of acromegaly and is recommended in patients previously treated by surgery or radiotherapy whose disease cannot be adequately controlled even with the maximum doses of SRLs (16). PEGV may be used as monotherapy or in combination with SRL and/or DA (17). The GH receptor antagonist may also be highly useful in acromegalic patients with poorly controlled diabetes mellitus in whom SRLs might worsen glucose metabolism (18).

The effectiveness of PEGV varies widely depending on the type of study (controlled vs. observational clinical trial) (19). In fact, IGF-1 levels returned to normal in over 90% of patients in controlled clinical trials (20,21), while the normalization rate was lower in observational trials (22)

In 2010, we published our first experience from a multicenter real-life study in our country, where we analyzed the outcome of 28 patients with acromegaly treated with PEGV for a mean time of 12 months (23). We found that disease control had been achieved in 58% of patients with a mean dose of PEGV of 9.6 mg/day (23). We reported a rate of adverse events similar to the rates observed in various publications. Therefore, we decided to continue analyzing these and new patients with the aim of obtaining further data on the effectiveness and safety of PEGV in the long-term treatment of acromegaly in real life data set

The aim of our study was to contribute further data to the national registry of patients initiated in 2010. This is a multicenter, retrospective, observational study whose main objective was to evaluate the efficacy and safety of PEGV in the long-term treatment of acromegaly in the clinical practice setting. A secondary objective was to search a predictor of treatment response with PEGV

## SUBJECTS AND METHODS

The study is not based on a protocol but conducted according to international algorithms and the reality of our healthcare system, whether public or private (real-life study).

Ambulatory patients from 17 sites in Argentina with a diagnosis of acromegaly, treated with PEGV for at least two months at any time during the course of their disease. Diagnosis of acromegaly was based on elevated age- and sex-adjusted IGF-1 levels and lack of GH suppression to < 1 ug/l during the oral glucose tolerance test (OGTT) (14).

Baseline data at the time of the diagnosis of acromegaly were collected: age, gender, GH and IGF-1 levels and tumor size (micro/macroadenoma). Data on various treatment regimens (surgery, radiotherapy, drug therapy), and size of tumor remnant prior to PEGV initiation were also registered. Finally, biochemical and imaging data during treatment with the GH receptor antagonist were analyzed.

PEGV treatment indication and monitoring were at the treating physician’s criterion.

IGF-1 levels were measured at local labs and interpreted according to each laboratory’s age- and gender-adjusted reference ranges.

IGF-1 results were expressed as the ratio of the absolute value to the upper normal limit of the reference method (IGF-1xULN). A cutoff point ≤ 1.2 x ULN as parameter of biochemical control to PEGV therapy was considered in this analysis.

As regards drug safety, tumor size and liver enzyme levels were evaluated before and after treatment with PEGV, as well as the occurrence of other adverse events such as local reactions at the site of injection.

Inform consent was obtained from all individual participants included in this study.

### Statistical analysis

For the descriptive analysis, categorical variables were expressed as the frequencies, percentages and 95% confidence intervals, and the numerical variables were expressed as the mean ± SD, 95% confidence intervals for mean. The Student test for paired samples was used to evaluate the differences between IGF-I levels before and after PEGV therapy. The ANOVA for repeated measures was applied to evaluate the differences in the follow up to the 3, 6 and last follow up. The Cochran’s Q test for sample paired with Bonferroni correction was used to compare proportions between follow ups. The difference was considered significant when p < 0.05.

## RESULTS

### Baseline characteristic

Seventy -five patients (51 women), age 41.96 ± 12.61 years (range 21-68) diagnosed with acromegaly between 1985 and 2012 were included.

Baseline IGF-1 (IGF-1/ULN) at the time of diagnosis of acromegaly was 3 ± 1.4 ULN (range 1.4 to 5.6).Of the 75 patients, 63 (84%) had images consistent with macroadenomas, and 10 (13.3%) with microadenomas. In 2 patients clinical and biochemical GH excess was documented, with no evidence of pituitary tumor or ectopic disease (Table 1).

**Table 1:** Baseline Patient Characteristics (n = 75)

Sex, % Females	68%
Age at diagnosis, years	41.9 (21-68)
IGF1 x ULN at diagnosis	3 ± 1.4 (range 1.4-5.6)
Tumor size at diagnosis % macro	84%
Previous Therapy	97.3%
Surgery + MT	40%
Surgery + RT + MT	37.3%
Medical Treatment alone	16%
RT alone	4%
No therapy	2.7%
Age prior PEGV years	47.5 (22-77)
IGF-1 xULN at start of PEGV	2.4 ± 0.98 (range 1.25-7)
Tumor size prior PEGV % macro	23.5%

Data are expressed as median or percentage.

MT: medical therapy; RT: radiotherapy.

### Previous treatments

Of the total of 75 patients, 30 (40%) were treated with surgery and drug therapy; 28 (37.3%) with surgery, radiotherapy and medical treatment; 12 (16%) only drug therapy; 3 (4%) radiotherapy and medical treatment prior to the start of PEGV. Some of the reasons why surgery was not considered as first option of treatment were: invasive tumors with low chance of cure, patients with severe comorbidities and high surgical risk, lack of a neurosurgical team with expertise in pituitary tumors, and patient rejection.

Two patients (2.7%) received PEGV as first line treatment (Table 1). One was a 67-year-old woman with a microadenoma at the diagnosis and severe chronic obstructive pulmonary disease who was contraindicated for using SRL due to gallbladder lithiasis. The other patient was a 58-year-old woman with a microadenoma as well, and severe hypertension who initiated primary therapy with PEGV assuming a faster and more effective control of IGF-1 excess.

Of the 73 patients receiving drug therapy prior to PEGV, 48 (65.8%) received SRL first generation in combination with cabergoline, while 25 (34.2%) were treated only with SRL.

### Pre-pegvisomant residual tumor size

Information on residual tumor size after multimodal treatments, and prior to PEGV therapy, was as following: 37,3% patients had no residual tumor (including 2 patients with normal MRI at the time of diagnosis), 39.2% and 23.5% showed residual tumor size < 1 cm and > 1 cm respectively.

### Treatment with Pegvisomant

The mean age of patients at the time of initiating treatment with PEGV was 47.5 years (22-77). Mean serum IGF-1 (IGF-1/ULN) levels prior to PEGV therapy were 2.4 ± ٠.٩٨ (range ١.٢٥ to ٧). The dose of PEGV prescribed ranged from 20 to 210 mg weekly. The mean dose was 11.8 mg/daily. Median treatment duration with PEGV was 27 months, with a range of 2 to 118 months.

Thirty-four patients out of 75 (45%) received monotherapy with PEGV, and 41 (55%) received combined therapy. Of the latter, 28 (68.3%) received SRL; 8 (19.5%) cabergoline with SRL, and the remaining 5 (12.2%) cabergoline.

### IGF-1 levels during treatment with pegvisomant

We considered IGF-1/ULN ≤ 1.2 as the cutoff point for referring to controlled disease.

Biochemical treatment response was evaluated in 62 of 75 patients. Thirteen patients were excluded from the treatment response analysis due to the lack of available or reliable IGF-1 levels, or early drug discontinuation.

**Figure 1:**
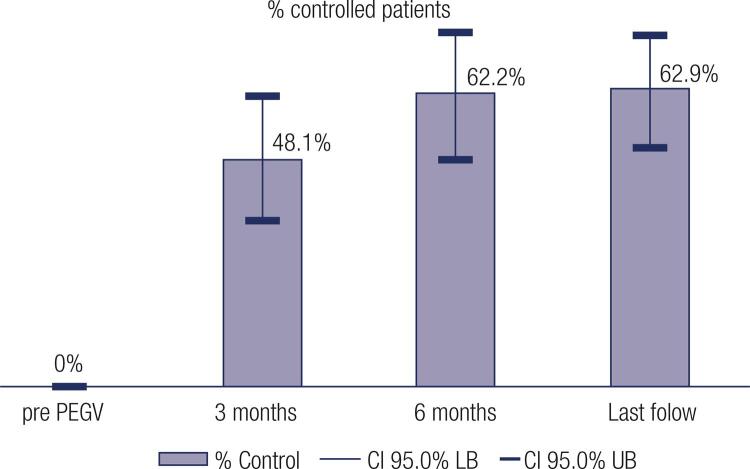
Response Rate of the 62 patients with acromegaly during follow up treatment with PEGV.

Disease control was achieved in 39 patients (62.9%) during the last follow up and were included into the “controlled group”: IGF-1 levels did not return to normal in 23 patients (36.9%), who were included into the “uncontrolled group” (Figure 1).

**Figure 2:**
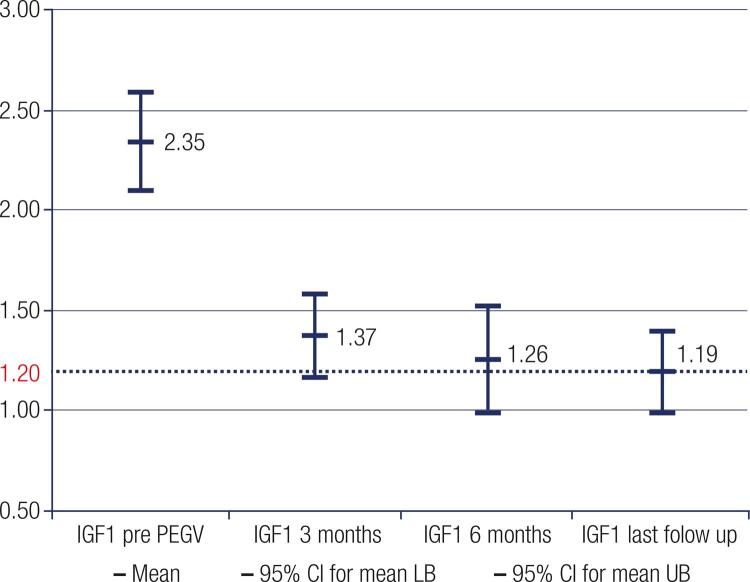
IGF-1 levels (IGF-1/ULNin 62 patients with acromegaly, before and during long term follow up treatment with PEGV.

Considering the total group of 62 patients, the mean pre-PEGV IGF-1 level (IGF-1/ULN) was 2.35 (95% IC 2.10-2.59), decreasing to 1.37 (95% IC 1.16-1.58), and 1.26 (95% IC 0.99-1.52), at 3, 6 months of treatment respectively and to 1.19 (95% IC 0.99-1.40), at the last follow-up, observing statistical differences between the pre-PEGV IGF-1 level and the other values (p < 0.001) (Figure 2).

**Figure 3:**
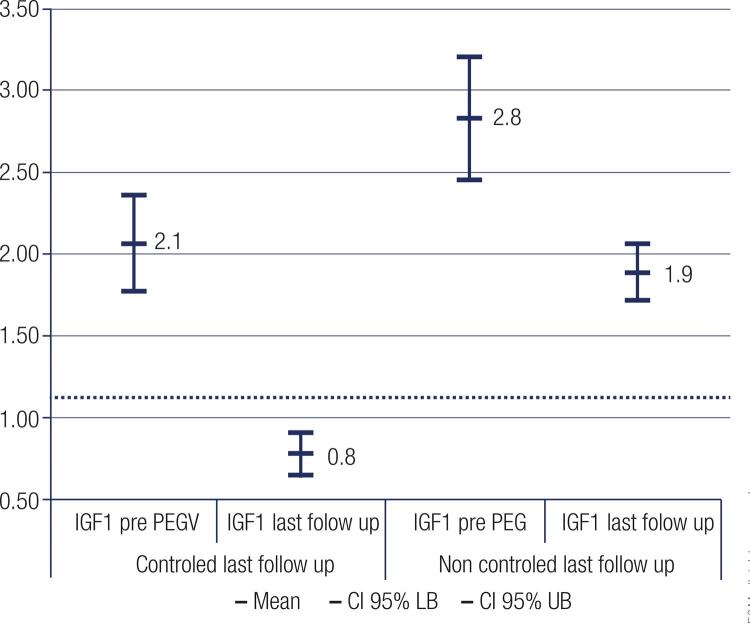
IGF-1 levels (IGF-1/ULN) in controlled vs uncontrolled patients with acromegaly before and at the last follow up treatment with PEGV.

When IGF-1 levels were analyzed in the different groups of patients according to biochemical response we obtained the following results: in controlled patients mean pre-PEGV IGF-1 levels decreased from 2.06 (95% IC 1.77-2.35), to 0.78 (95% IC 0.65-0.91) (p < 0.0001), in uncontrolled patients mean pre-PEGV IGF-1 decreased from 2.83 (95% IC 2.45-3.21), to 1.89 (95% IC 1.72-2.06), at the last follow-up (p < 0.0001) (Figure 3).

Regarding modality of treatment, there were normalization of IGF-1 levels in 64.7% and 60.7% of combined and monotherapy treatments respectively, with no significant difference. Considering cutoff levels of IGF-1 x ULN ≤ 1, 58% of patients were defined as controlled under PEGV treatment.

### Predictors of response

There was no difference in age, gender, previous radiotherapy or pre-PEGV GH levels between patients who were controlled and those who were not after PEGV treatment When comparing mean pre-PEGV IGF-1levels in the group of patients who achieved disease control vs. the group of patients with uncontrolled disease, we found significant lower mean levels in the controlled group: 2.1 vs. 2.8; (p < 0.001).

### Dose of pegvisomant

The mean dose of PEGV used in the whole group was 11.5 mg/day. The group of patients not achieving disease control received an average dose of 13.9 mg/d (median 15, range 6-30), higher than that received by the group with controlled disease (mean 10.6 mg/d, median 10, range 3-20) (p = 0.046).

We did not find significant differences between the dose of PEGV prescribed as combined therapy and the one prescribed as monotherapy: 11.4 mg/d (SD = 53) vs. 11.4 mg/d (SD = 5.6), respectively (p = 0.99).

Regarding uncontrolled patients, most of them were prescribed to increase the dose of PEGV, however due to the lack of social security support, adverse events, or lack of compliance, dose optimization was not completely fulfilled.

### Safety

Sixteen patients experienced an adverse event related to PEGV.

#### Local adverse events

Four out of 75 patients (5.3%) experienced local adverse events: 3 had localized lipodystrophy and 1 cellulitis. Of these 4 patients, 2 required PEGV discontinuation.

#### Hepatotoxicity

Increased transaminase levels (> 3X ULNwas reported in 7 of 75 (9%) patients. Following treatment discontinuation or dose reduction, transaminase values returned to normal in all patients. Two patients reinitiated treatment, at this time with no enzyme elevation. The five remaining patients did not reinitiate treatment with PEGV: 3 as a precaution, 1 by decision of the patient and 1 due to drug unavailability. One of the patients was previously published (24).

#### Tumor growth

**Figure 4:**
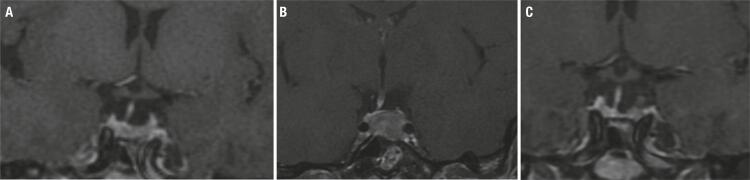
Coronal T1 weighted image after gadolinium. (A) Tumor remnant on the left side of the pituitary gland before PEGV (after 1st surgery, RT and SRL). (B) Tumor growth After 72 months PEGV monotherapy. (C) Disease remission and no tumor remnant after 2nd surgery.

Four out of 50 patients (8%) with available images, showed an increase in tumor size on PEGV. Three patients (2 macroadenomas, 1 microadenoma) had been operated on and received SRL prior to the use of PEGV, one of them with elevated Ki 67 on tumor pathology, had also undergone gamma knife radiotherapy (Figure 4). Both patients with macroadenomas required subsequent surgery due to tumor growth under PEGV; one of them, additional radiotherapy as well. The patient with a microadenoma returned to SRL therapy after 1 year PEGV, with tumor shrinkage in the long term follow up. The fourth patient started PEGV as first line treatment, due to severe comorbidities and partial contraindication to surgery and SRL; the tumor increased from 7 to 11 mm after 1year PEGV. After that term the patient became lost to follow-up. All four patients achieved normal or reduced levels of IGF-1 under PEGV treatment, three of them on monotherapy and one on combination therapy with SRL. It should be noted that the images (MRI) were not evaluated by a central team.

PEGV discontinuation due to serious adverse events was documented in 6 patients (3 local reaction, 2 hepatotoxicity and 1 tumor enlargement). Some patients discontinued therapy as a precaution or due to lack of PEGV availability.

## DISCUSSION

PEGV was approved for use in acromegaly in Argentina in 2005. The present study is the largest Latin American series of patients with acromegaly that shows long-term treatment outcome with PEGV.

We demonstrated that PEGV treatment is effective and safe in our cohort of Argentine patients with acromegaly similar to that observed in other populations. We have expanded the national registry of patients with acromegaly treated with PEGV, from our first experience in 2010 when we analyzed the outcome of 28 patients with acromegaly treated with PEGV for a mean treatment duration of 12 months, achieving IGF-1 normalization in 58.8 % of them (23).

Acromegaly is associated with increased mortality and morbidity when levels of GH and IGF-I fail to normalize (25). Although surgery and tumor-targeted drugs allow disease control in most patients, there are some cases that require additional treatments. In our patient series, 77.3% had undergone previous surgery, 97.3% had received first-generation SRLs alone or in combination with cabergoline, and 41.3% received radiotherapy prior to the initiation of PEGV. There were only two patients who initiated PEGV as primary treatment.

PEGV effectiveness in normalizing IGF-1 levels in patients with acromegaly is highly variable. In controlled prospective studies, normalization of IGF-1 levels is observed in up to 97% of patients (20] while in observational studies based on daily clinical practice, such normalization is lower (26,27). Acrostudy is the largest international study of acromegalic patients treated with PEGV conducted since 2004 to the present. The first and second interim analysis of this study included data collected on 1288 and 2090 patients respectively, obtaining disease control in 63% to 73% of them (26,27)

Multiple factors contribute to the discrepancy in IGF-1 control between clinical trials and observational studies: the absence of a dose titration scheme according to each patient’s needs (typically done in clinical trials), the use of different criteria for disease control, the use of combined treatments schemes, the centralized review of data in controlled clinical trials (such as laboratory values and images), the regularity of follow-up of the individuals evaluated, and patients treatment compliance. Notably, non-controlled observational studies usually include patients who have not achieved acromegaly control despite multiple treatments, often those with more aggressive disease and probably more comorbidities, thus leading to a bias in patient enrollment (26).

We considered IGF-1/ULN ≤ 1.2 as the cutoff point for referring to controlled disease, according to our day to day clinical practice, since below this cutoff level we do not change therapeutic decisions. On the other hand, there is enough evidence supporting the use of this cutoff value in acromegalic patients monitored under medical treatment with PEGV or LRS (28).

We demonstrated that PEGV was effective in normalizing IGF-1 in 62.9% of patients, with a mean treatment duration of 27 months. We expanded the database of patients with acromegaly treated with PEGV started in 2010.

These results are better to those obtained in our first analysis, possibly due to the use of higher mean doses of PEGV (11.5 mg/day vs 9.6 mg/day respectively) and longer follow up.

Recently, a Brazilian study (29) published the findings from a series of 27 patients retrospectively evaluated, reporting 85% normalization of IGF-1 under PEGV treatment. The authors attribute this high rate of effectiveness to the fact that they are tertiary reference center with high experience in treating acromegaly and therefore, patients have a more appropriate dose adjustment to attain the goals of disease control. The median dose of PEGV was 10 mg/day in the controlled patients, whereas it was 22.5 mg/day in the uncontrolled patients. In our study the median doses were 10 and 15 mg/day for the controlled and uncontrolled patients respectively, suggesting the lack of optimization in the latest group which didn´t reach the maximal allowed daily dose according to the label. It is remarkable the significant decrease of IGF-1 in the uncontrolled group, in spite of not achieving a safe IGF-1. This rate of reduction in IGF-1 levels may improve associated comorbidities of patients with partial controlled acromegaly, and as a consequence enhance their quality of life.

On the other hand the greater use of combined therapy with PEGV and SRL might improve the number of controlled patients, as shown by the Brazilian study (74% combined treatment) and previous reports (30,31). In our series, 55% of patients were on combination therapy, which could possibly be not enough to get a better rate of response.

Regarding the safety of PEGV, we found that the increase in liver enzymes associated with the use of PEGV was 9%, similar to 7.7% and 7.4% reported by the French and Brazilian groups, respectively (29,32). Other investigators reported higher rate of hepatotoxicity for combined treatment with SRL (30).

Tumor growth under PEGV treatment in our study was 8%, similar to that reported by Grottoli and cols. in 2015 (8.8%) (33). Our study, as in most observational studies, did not use a central team for reviewing the images. Buchfelder and cols. demonstrated in a large group of PEGV-treated patients, that tumor progression was rare. Out of 307 patients, 18 were reported to have tumor-size increases as adverse events, but only 3 were considered true growth after the re-evaluation. They recommend a careful serial evaluation of all available images to avoid misinterpretations (34).

There are few studies in the literature addressing predictors of response to PEGV treatment (35). We found that the group of controlled patients had significant lower pre-PEGV IGF-1 levels than the group of patients not achieving disease control. Therefore, lower baseline IGF-1 levels before the initiation of PEGV might predict a better response to PEGV treatment, in agreement with findings reported by the Brazilian group (29).

The limitations of the study might be the low number of patients compared to the international series, the lack of optimization of the PEGV treatment with higher doses in the uncontrolled group, and the lack of a central team for the evaluation of the images. However, it has the strength to be the largest series of PEGV treatment in acromegaly in Latin America, in the setting of a real life data.

In conclusion, this observational, multicenter study for the evaluation of the response to PEGV treatment in 75 patients with acromegaly in Argentina demonstrates that PEGV is an effective and safe drug for the treatment of uncontrolled acromegaly, with IGF-1 normalization rates similar to those reported in the international literature. The significant decrease in IGF-1 levels is ultimately associated with a reduction in acromegaly comorbidities, an improvement in the quality of life and prolonged life expectancy
